# Ice swimming and changes in body core temperature: a case study

**DOI:** 10.1186/s40064-015-1197-y

**Published:** 2015-08-05

**Authors:** Beat Knechtle, Thomas Rosemann, Christoph A Rüst

**Affiliations:** Institute of Primary Care, University of Zurich, Zurich, Switzerland; Gesundheitszentrum St. Gallen, Vadianstrasse 26, 9001 St. Gallen, Switzerland

**Keywords:** Open-water swimming, Ice swimming, Body fat

## Abstract

**Introduction:**

‘Ice Mile’ swimming is a new discipline in open-water swimming introduced in 2009. This case study investigated changes in body core temperature during preparation for and completion of two official ‘Ice Miles’, defined as swimming 1.609 km in water of 5°C or colder, in one swimmer.

**Case description:**

One experienced ice swimmer (56 years old, 110.2 kg body mass, 1.76 m body height, BMI of 35.6 kg/m^2^, 44.8% body fat) recorded data including time, distance and body core temperature from 65 training units and two ‘Ice Miles’.

**Discussion and evaluation:**

During training and the ‘Ice Miles’, body core temperature was measured using a thermoelectric probe before, during and after swimming. During trainings and the ‘Ice Miles’, body core temperature increased after start, dropped during swimming but was lowest during recovery. During training, body core temperature at start was the only predictor (ß = −0.233, p = 0.025) for the increase in body core temperature. Water temperature (ß = 0.07, p = 0.006) and body core temperature at start (ß = −0.90, p = 0.006) explained 61% of the variance for the non-significant decrease in body core temperature. Water temperature (ß = 0.077, p = 0.0059) and body core temperature at finish (ß = 0.444, p = 0.02) were the most important predictors for the lowest body core temperature. In ‘Ice Miles’, body core temperature was highest ~6–18 min after the start (38.3–38.4°C), dropped during swimming by 1.7°C to ~36.5°C and was lowest ~40–56 min after finish. The lowest body core temperature (34.5–35.0°C) was achieved ~100 min after start.

**Conclusions:**

In an experienced ice swimmer with a high BMI (>35 kg/m^2^) and a high percent body fat (~45%), body core temperature decreased by 1.7°C while swimming and by 3.2–3.7°C after the swim to reach the lowest temperature in an official ‘Ice Mile’. The swimmer suffered no hypothermia during ice swimming, but body core temperature dropped to <36°C after ice swimming. Future athletes intending to swim an ‘Ice Mile’ should be aware that a large body fat prevents from suffering hypothermia during ice swimming, but not after ice swimming.

## Background

Open-water swimming is of high popularity (Eichenberger et al. 2012). Apart from the ‘English Channel Swim’ held since 1875 (Knechtle et al. 2014), competitive open-water ultra-distance swimming is held at world class level since 2000 for different distances such as 5, 10 and 25 km (Vogt et al. 2013; Zingg et al. 2014).

A very young and new discipline in open-water swimming is the so-called ‘ice mile swimming’. In 2009, Ram Barkai founded the International Ice Swimming Association (IISA) in South Africa (http://www.internationaliceswimming.com). The IISA introduced the ‘Ice Mile’ as its ultimate achievement of swimming in ice cold water where an ‘Ice Mile’ is defined as swimming one mile (1.609 km) in water of 5°C or colder. The swim must be unassisted and only with one pair of swimming goggles, swimming cap and standard swimming suit. In addition to the ‘Ice Mile’, the IISA introduced in 2014 the ‘1 km Ice event’. Since 2009 and 2014, swim times in ‘Ice Mile’ and ‘1 km Ice event’, respectively, are recorded.

In ice swimming, safety implications and the avoidance of hypothermia and non-freezing cold injury are key success factors. Issues pertaining to cold shock, drowning and circum-rescue collapse are highly relevant in this population. To date, only one case study has been published where two swimmers were presented while swimming in ice cold water (Knechtle et al. 2009). However, in that case, body core temperature was not measured during ice swimming, but only before and after swimming. One successful swimmer presented a body core temperature of 32°C after swimming 2.2 km in water of 4°C (Knechtle et al. 2009).

The aim of this actual case report was to present changes in body core temperature in an experienced ice water swimmer during training swims and swimming two official ‘Ice Miles’. Based upon the findings in the case report of swimming 2.2 km in ice water, we hypothesized that the swimmer would suffer hypothermia (body core temperature <36.0°C) during ice swimming (Nuckton et al. 2000). We were also interested in body core temperature after ice swimming to find a potential after drop. These findings might be of help for future athletes intending to swim an ‘Ice Mile’.

## Methods

All procedures applied in the study were approved by the Institutional Review Board of Kanton St. Gallen and the athlete gave his written informed consent. Additional informed written consent was obtained from the participant for whom identifying information is included in this article.

### Participant

An experienced open-water ultra-swimmer (56 years old, 110.2 kg body mass, 1.76 m body height, BMI of 35.6 kg/m^2^) participated in the study. The swimmer has a broad experience in open-water ultra-swimming. For example, in 2011, he was the first swimmer ever to cross the Fehmarn-Belt from Fehmarn (Germany) to Rødby (Denmark) and back to Fehmarn (i.e. double crossing) (http://www.bruno-dobelmann.de). In 2012, he was swimming in water colder than 10°C for 6 h (Rüst et al. 2012). The subject was healthy, had no medication and no history of non-freezing cold injury such as loss of sensation, skin damage/infection, or nerve damage.

### Preparation for the ice mile swimming

In the preparation for the two ‘Ice Miles’, the athlete completed weekly 45–85 swimming kilometers. He stopped in 2012 swimming in indoor and outdoor pools and performed all swim trainings in open water (i.e. lakes) all the year round independent of weather and temperatures. Several trainings were performed in lakes where the distance could not be measured. Other trainings were held in small lakes where lanes were set and laps were counted by the support crew to determine the covered distance. He started specifically preparing for the ‘Ice Miles’ in September 21, 2012, and recorded for specific swim trainings (i.e. in ice cold water and/or longer trainings) the distances, the times and the water and air temperatures. In all recorded training units, body core temperature was continuously measured before, during and after the swim (Table 1). Between September 2012 and January 2015, body mass remained stable at ~112–115 kg.

**Table 1:** Data from the trainings prior to the ice mile swimming

Date	Distance (km)	Time (h:min)	Speed (km/h)	Water temperature (°C)	Air temperature (°C)	Temperature at start (°C)	Highest temperature (°C) (h:min after start)	Temperature at finish (°C)	Lowest temperature (°C) (h:min after finish)
21/09/2012	6.0	02:12	2.72	16.6	10.0			38.2	
25/09/2012	10.0	03:46	2.65	16.9	16.4	37.4		38.3	
27/09/2012	6.0	02:17	2.62	16.6	12.2	37.3		38.2	
10/10/2012	3.0	01:10	2.57	14.3	16.1	37.4	37.8 (0:17)	37.7	
11/10/2012	5.0	01:50	2.72	13.8	15.6	37.2	38.0 (0:48)	38.0	
22/10/2012	4.0	01:35	2.52	13	12.1	37.4	37.9	37.8	
25/10/2012	4.0	01:30	2.66	12.1	9.9	37.6	38.0	37.7	
30/10/2012	2.4	00:50	2.88	9.0	8.6	37.7	38.1	38.0	
01/11/2012	2.6	01:00	2.60	8.9	11.2	37.6	37.9 (0:22)	37.4	36.4 (0:58)
05/11/2012	3.0	01:15	2.40	10.0	9.7	37.5	37.9 (0:39)	37.7	36.5 (1:36)
09/11/2012	3.0	01:14	2.43	9.0	12.0	37.6	37.9 (0:25)	37.2	36.8
10/11/2012	3.4	01:25	2.40	8.7	11.0	37.8	38.0 (0:12)	37.0	36.2 (0:43)
13/11/2012	2.6	01:00	2.60	8.7	11.0	37.6	38.0 (0:32)	37.7	36.4 (1:37)
15/11/2012	3.0	01:14	2.43	8.4	11.0	37.6	37.7 (0:30)	37.3	36.1 (1:26)
17/11/2012	2.8	01:00	2.80	7.8	8.0	37.6	38.1	37.2	36.4
19/11/2012	2.0	00:41	2.92	7.4	9.8	37.5	37.7	37.4	36.2
21/11/2012	1.0	00:22	2.72	7.1	19.0	37.6	37.7	37.6	36.4
09/12/2012		00:35		6.0	−8.0	37.9	38.2	37.7	36.4
26/12/2012	0.6	00:23	1.56	5.0	12.0	38.3	38.6	38.5	37.7
30/12/2012	0.8	00:28	1.71	4.4	9.0	37.6	37.7	37.2	36.2
03/01/2013	0.8	00:18	2.66	4.0	7.4	37.8	38.1 (0:16)	38.0	36.2 (1:04)
06/01/2013	1.6	00.45	2.13	4.9	7.0	37.9	38.1 (0:11)	37.0	35.7 (0:40)
09/01/2013	1.4	00:30	2.80	4.7	6.0	37.4	37.5 (0:08)	36.4	35.6 (0:38)
13/01/2013	1.0	00:25	2.40	3.9	0.5	37.8	37.9	37.7	36.0
16/01/2013		00:32		1.4	0.7	37.8	38.0	37.5	36.1
19/01/2013		00:37		1.7	0.3	38.1	38.4	37.8	36.3
26/01/2013		00:32		0.9	0.5	38.1	38.1 (0:21)	37.5	36.1 (1:03)
27/01/2013		00:35		1.1	3.0	37.8	38.1 (0:13)	37.7	35.9 (0:35)
30/01/2013		01:00		1.7	16.0	37.8	38.0 (0:09)	35.9	34.6 (0:51)
02/02/2013	2.2	01:01	2.16	4.6	4.3	38.2	38.3 (0:15)	36.5	35.8 (0:35)
07/02/2013		00:27		1.9	3.3	37.7	37.9 (0:10)	37.4	36.3 (0:33)
10/02/2013		01:02		2.2	−3.6	37.7	37.9 (0:11)	36.0	34.6 (0:45)
17/02/2013		01:01		2.3	3.5	38.0	38.1	36.8	35.7
20/02/2013		01:19		3.2	4.2	37.8	37.8	36.2	35.2
23/02/2013		00:47		1.7	−2.6	37.9	37.9	36.6	35.4
24/02/2013		00:50		1.6	−1.7	37.8	37.9 (0:10)	36.4	35.0 (0:45)
02/03/2013		00:51		2.5	4.5	38.0	38.0	36.9	35.7 (0:33)
04/04/2013	1.05	0:45	1.40	1.5	0	38.0	38.1 (0:11)	36.9	35.4 (0:47)
30/08/2013	13.0	5:03	2.57	21.0	27.0	37.1	38.2 (0:42)	38.4	37.4 (1:28)
31/08/2013	7.0	2:39	2.64	20.1	25.5	36.9	38.1 (0:56)	37.8	37.0 (2:16)
01/09/2013	12.0	4:38	2.58	20.7	25.0	37.1	37.9 (0:42)	37.8	37.0 (0:57)
03/09/2013	6.0	2:10	2.76	19.9	14.5	37.6	38.1 (0:52)	38.1	37.1 (1:40)
13/10/2013	2.5	1:58	1.27	12.1	14.0	37.7	37.7	38.2	37.2 (1:10)
20/10/2013		1:30		12.2	16.0	37.7	38.4 (1:06)	38.3	37.2 (1:22)
22/10/2013		1:25		13.6	23.0	37.5	38.2 (1:14)	38.1	36.9 (1:28)
17/11/2013	2.0	0:44	2.72	7.7	8.0	37.9	38.0 (0:04)	36.9	36.6 (0:26)
18/11/2013	2.0	0:45	2.66	7.6	2.0	37.9	38.0 (0:06)	36.9	36.6 (0:28)
23/11/2013		0:40		6.3	6.0	37.8	38.0 (0:14)	37.1	36.2 (0:44)
30/11/2013	0.6	0:20	1.80	4.0	4.3	37.5	37.5	37.0	35.8 (0:36)
08/12/2013		0:46		4.9	5.0	37.9	38.1 (0:26)	37.8	36.7 (0:50)
15/12/2013		0:30		4.3	5.5	37.7	37.9 (0:14)	37.2	35.7 (0:24)
22/12/2013		0:40		4.0	11.0	37.9	38.1 (0:14)	36.9	35.9 (0:40)
28/12/2013	0.8	0:20	2.40	2.3	6.0	37.7	37.8 (0:08)	37.5	35.5 (0:34)
11/05/2014	8.0	3:00	2.66	16.5	18.0	37.4	38.0 (0:58)	37.9	36.9 (2:04)
18/05/2014	9.0	3:20	2.70	16.4	16.0	37.6	38.4 (1:22)	37.5	37.1 (1:36)
24/05/2014	8.0	3:10	2.52	20.5	19.0	37.3	38.2 (1:12)	38.0	36.6 (2:40)
25/05/2014	4.0	1:16	3.157	19.4	18.0	37.8	38.3 (0:34)	38.1	37.1 (1:46)
30/10/2014	3.0	1:12	2.50	13.3	14.6	37.4	38.0 (0:46)	37.9	37.1 (1:50)
01/11/2014	3.0	1:09	2.60	9.8	13.4	37.8	38.2 (0:36)	38.1	37.0 (1:20)
02/11/2014	3.65	1:30	2.43	8.4	12.0	37.2	37.8 (0:48)	37.8	
11/11/2014	3.0	1:09	2.60	9.8	13.4	37.8	38.2 (0:35)	38.1	37.0 (2:15)
20/11/2014	1.8	0:46	2.34	10.2	8.9	37.4	37.7 (0:26)	37.5	36.2 (1:28)
02/12/2014		0:22		5.0	3.0	37.8	38.1 (0:13)	38.1	36.7 (0:36)
01/01/2015		0:10		5.1	4.0	38.0	38.0	38.0	37.5 (0:26)
10/01/2015		0:15		2.2	1.8	37.3	37.5 (0:12)	37.5	36.7 (0:32)

### Procedures

For the trainings, we determined body core temperatures at the start and the finish of the swims from the recorded data, and the lowest and the highest body core temperatures during the recorded time. For the ‘Ice Miles’, prior to the start of the first ‘Ice Mile’, we measured body composition using Dual-energy X-ray absorptiometry (DXA) (Lunar iDXA™, GE Healthcare, Madison, WI, USA) to determine body fat percentage and lean body mass. Body core temperature was measured continuously in the rectum using the thermoelectric probes Endotherm^®^ (EndoTherm GmbH, Arlesheim, Switzerland) before the start, during the swim and after the swim while recovering. The probe was inserted in the rectum using a protective container provided by the manufacturer. Endotherm^®^ measures temperatures from −40 to +85°C with a resolution of 0.0625°C and a precision of 0.1°C. The Endotherm^®^ probes were programmed to take one measurement every 12 s (i.e. five measurements per minute) and were applied before the start of the swims. In case a temperature (e.g. body core temperature at the start and the finish, and the lowest and the highest body core temperature during the recorded time) remained stable for more than one measurement, we took that time when the temperature appeared first. During all trainings and both ‘Ice Miles’, the athlete wore their swimming trunks and their swimming googles. After the swims in water of 5°C and colder, the athlete wore a thermal blanket during recovery while he was shivering during the afterdrop.

### Data analysis and statistical analysis

All statistical analyses were performed using Analyse-it (Analyse-it Software Ltd, The Tannery, 91 Kirkstall Road, Leeds, LS3 1HS, United Kingdom). Statistical significance was set at *p* < 0.05. We calculated effect sizes since the use of *p* values provides no information about the direction or the size of the effect (Hopkins et al. 2009). Effect sizes were classified as trivial (<0.2), small (0.2–0.6), moderate (0.6–1.2), large (1.2–2.0), very large (2.0–4.0) and extremely large (> 4.0) (Hopkins 2006). To investigate potential differences between measured body core temperatures (i.e. temperature at start, temperature at finish, lowest and highest temperature), a paired *t* test was used. Cohen’s *d* was used to indicate the standardized difference between two means. We performed correlation analyses to find potential associations between water temperature, swim distance and changes in body core temperature. Since all data were normally distributed, Pearson correlation analysis was used. To calculate effect sizes of the correlations, we used the equation $2 \times {\text{r}}/\sqrt {1 - {\text{r}}^{2} }$. In case we found associations between variables, we applied multi-variate regression analyses to find the most predictive variables. Cohen’s *ƒ*^2^ was used to measure the effect size measures in the context of a multiple regression.

## Results

### Pre event from 2012 to 2015

In the DXA, the athlete had 44.8% body fat, 48.08 kg fat mass, 59.15 kg lean body mass and 2.92 kg bone mineral content. Table 1 presents the 65 trainings units with date, distance (if available), time, swimming speed (if available), body core temperatures at the start and at the finish and the highest and the lowest body core temperatures (if available). For 43 trainings, swim distance could be determined. On average, he was swimming for 3.78 ± 3.04 km (range 0.6 to 13 km) at a mean speed of 2.47 ± 0.38 km/h (range 1.27–3.15 km/h). Water temperature and swimming speed correlated significantly and positively (r = 0.43, *p* = 0.0045).

**Fig. 1:**
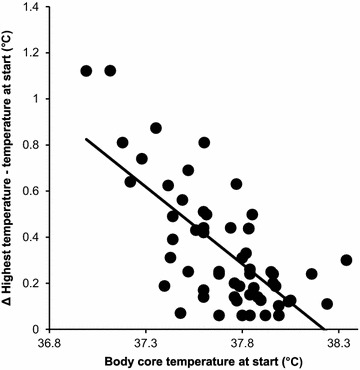
Relationship between body core temperature at start and the increase in body core temperature during the swim.

Body core temperature increased after the start of the swims. At the start, body core temperature was 37.7 ± 0.2°C (range 36.9–38.3°C) and increased within 29.3 ± 20.9 min (range 4.0–82 min) by 0.3 ± 0.2°C (range 0.06–1.12°C) (*p* < 0.0001) to reach 38.0 ± 0.2°C (37.5–38.6°C) as the highest body core temperature (Cohen’s d −1.30, effect size r −0.54, small effect). The increase in body core temperature was significantly and positively related to water temperature (r = 0.85, *p* < 0.0001) (effect size 11.33, extremely large effect), the covered swim distance (r = 0.85, *p* < 0.0001) (effect size 11.33, extremely large effect), and overall swim time (r = 0.81, *p* < 0.0001) (effect size 8.52, extremely large effect). There was a significant and negative relationship (r = −0.70, *p* < 0.0001) (effect size 0.82, moderate effect) between the increase in body core temperature during the swims and body core temperature at the start. In the multi-variate analysis, body core temperature at the start was the only predictor variable (ß = −0.233, *p* = 0.025) (Cohen’s *ƒ*^2^ 0.96, moderate effect) for the significant increase in body core temperature and explained 80% of the variance (Fig. 1).

During the swims, body core temperature decreased non-significantly (p = 0.058) by −0.1 ± 0.7°C (range −1.9 to +1.3°C) to reach 37.5 ± 0.6°C (range 35.9–38.5°C) by the end of the swims (Cohen’s d 0.40, small effect). The non-significant decrease was significantly and positively related to the achieved distance (r = 0.61, *p* < 0.0001) (effect size 3.12, very large effect), swim time (r = 0.51, *p* < 0.0001) (effect size 2.08, very large effect), and water temperature (r = 0.72, *p* < 0.0001) (effect size 5.14, extremely large effect). Body core temperature at start was significantly and negatively related (r = −0.63, *p* < 0.0001) (effect size 3.49, very large effect) to the decrease in body core temperature during the swims. In the multi-variate analysis, water temperature (ß = 0.07, *p* = 0.006) (Cohen’s *ƒ*^2^ 1.08, moderate effect) and body core temperature at the start (ß = −0.90, *p* = 0.006) (Cohen’s *ƒ*^2^ 0.66, moderate effect) were the most predictive and explained 61% of the variance for the non-significant decrease in body core temperature during the swims.

The lowest body core temperature of 36.3 ± 0.6°C (range 34.6–37.7°C) was measured 65.9 ± 35.0 min (range 24.0–160.0 min) after the finish of the swims. The decrease of −1.1 ± 0.3°C (range −0.3 to −2.0°C) from the finish of the swims to the point in time of the lowest temperature was significantly and negatively related to swim time (r = −0.28, *p* = 0.03) (effect size 0.77, moderate effect) and water temperature (r = −0.37, *p* = 0.005) (effect size 1.17, large effect).

**Fig. 2:**
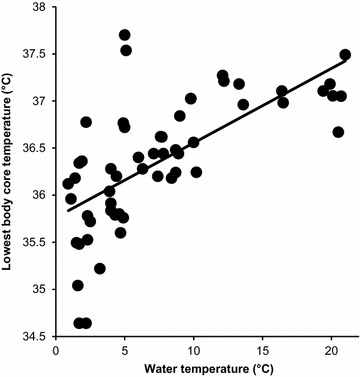
Relationship between water temperature and the lowest body core temperature.

**Fig. 3:**
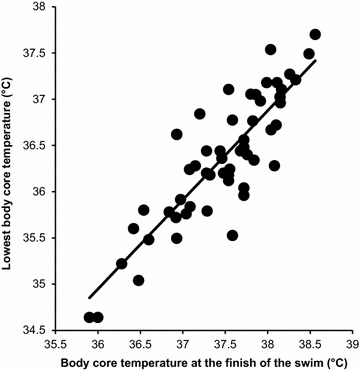
Relationship between body core temperature at the finish of the swim and the lowest body core temperature.

The lowest body core temperature was significantly and positively related to the covered swim distance (r = 0.56, *p* = 0.0007) (effect size 2.54, very large effect), the time spent in water (r = 0.40, *p* = 0.0024) (effect size 1.33, large effect), water temperature (r = 0.67, *p* < 0.0001) (effect size 4.06, extremely large effect), body core temperature at the end of the swim (r = 0.86, *p* < 0.0001) (effect size 12.28, extremely large effect), and the time from the swim start to the time when the lowest body core temperature was measured (r = 0.52, *p* = 0.0005) (effect size 2.16, very large effect). In the multi-variate analysis, water temperature (ß = 0.07, *p* = 0.0059) (Cohen’s *ƒ*^2^ 0.78, moderate effect) (Fig. 2) and body core temperature at the finish (ß = 0.444, *p* = 0.02) (Cohen’s *ƒ*^2^ 2.84, very large effect) (Fig. 3) were the most predictive and explained 77% of the variance.

**Fig. 4:**
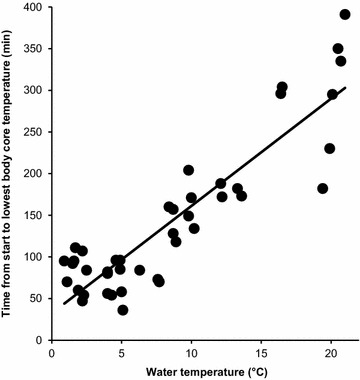
Relationship between water temperature and the time from the swim start to the lowest body core temperature.

We looked for associations between the time of the swim start and the time when the lowest body core temperature was achieved (143.0 ± 91.0 min, range 36.0–391.0 min). We found a significant and positive association with the covered distance (r = 0.94, *p* < 0.0001) (effect size 31.33, extremely large effect), the completed swim time (r = 0.95, *p* < 0.0001) (effect size 38.0, extremely large effect), water temperature (r = 0.90, *p* < 0.0001) (effect size 18, extremely large effect) and the body core temperature at the finish (r = 0.46, *p* = 0.002) (effect size 1.70, large effect). The time between the start of the swim and when the lowest body core temperature was achieved was significantly and negatively related to body core temperature at the start (r = −0.72, p < 0.0001). In the multi-variate analysis, water temperature (ß = 3.898, *p* = 0.049) (Cohen’s *ƒ*^2^ 4.0, very large effect) was the only predictor and explained 95% of the variance (Fig. 4).

### The two ‘Ice Miles’

**Fig. 5:**
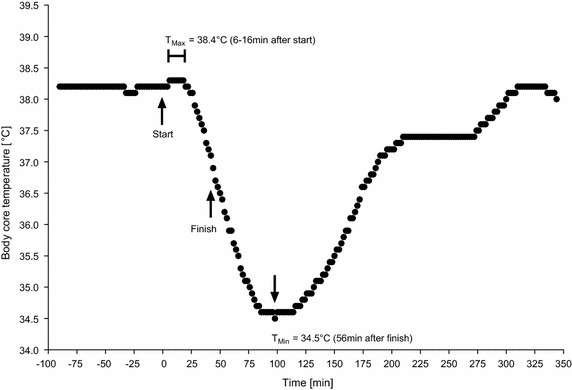
Change in body core temperature during the first ice mile.

**Fig. 6:**
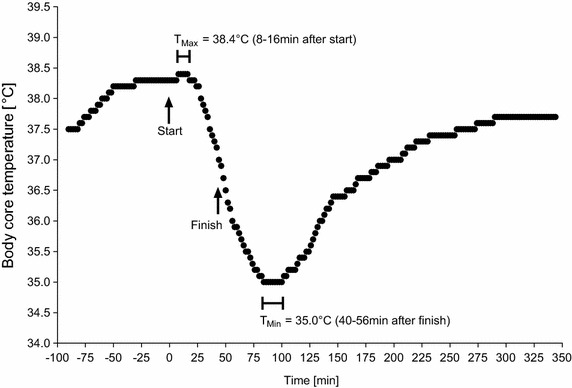
Change in body core temperature during the second ice mile.

The athlete completed the first ‘Ice Mile’ on December 31, 2014, at a water temperature of 4.8°C and an air temperature of 0°C within 0:44 h:min while swimming at a mean speed of 2.19 km/h. The second ‘Ice Mile’ was completed on February 22, 2015, at a water temperature of 3.9°C and an air temperature of 2.3°C within 0:41 h:min at a mean swimming speed of 2.35 km/h. Figures 5 and 6 show the changes in body core temperature for the first and the second ‘Ice Mile’, respectively. In the first ‘Ice Mile’, body core temperature was 38.2°C at the start, increased to a maximum of 38.3°C during the swim, dropped to 36.5°C by the end of the swim and dropped further to 34.5°C to the lowest temperature in the recovery. In the second ‘Ice Mile’, body core temperature was 38.2°C at the start, increased to a maximum of 38.4°C while swimming, dropped to 36.5°C at the end of the swim and dropped further to 35.0°C (lowest temperature) during recovery. Similarly to the trainings, body core temperature was highest ~6–18 min after the swim start, dropped continuously during the swim and was lowest ~40–56 min after the finish. On average, the lowest body core temperature was achieved ~100 min after the start in the first ‘Ice Mile’ and ~81–107 min after the start in the second ‘Ice Mile’.

## Discussion

This case study investigated changes in body core temperature in an experienced ice swimmer during preparation for and completion of two official ‘Ice Miles’. The most important findings were (1) body core temperature increased after the start of the swim (2) the swimmer suffered no hypothermia during the swims, and (3) the lowest body core temperature was measured ~45–60 min after the finish of the swims.

### No hypothermia during the swims

A first important finding was that body core temperature increased within the first few minutes after the start of the swims. The swimmer suffered no hypothermia during the swims, defined as body core temperature <36.0°C applying the American Heart Association definition of hypothermia (Nuckton et al. 2000). Body core remained unchanged during the training swims. The unchanged body core temperature was most probably due to his high body fat. In the case study of Knechtle et al. (2009), the successful swimmer who completed 2.2 km in water of 4°C had a body core temperature of 32°C after getting out of the water. The body fat percentage of the swimmer was only 23.4%, compared to the 44.8% of the present swimmer with the lowest body core temperature of 34.5°C after the first ‘Ice Mile’. The difference of ~2.5°C in body core temperature is very likely due to the ~21% difference in body fat percentage.

Body fat seems also to have an influence of staying in ice cold water. In swimmers competing in water of 11°C and colder, swimmers with less subcutaneous fat terminated their swims after significantly less time than athletes with thicker skinfold thicknesses (Keatinge et al. 2001). Longer swimming times in men with thicker skinfolds (i.e. with more body fat) were largely due to their greater buoyancy enabling them to keep their heads above water during the early hyperventilation (Keatinge et al. 1969). In a case description where two swimmers attempted to swim 2.2 km in water of 4°C, only the swimmer with more body fat (23.4 versus 21.0%) was able to reach the finish (Knechtle et al. 2009). High body fat has also been reported to increase survival in open water after shipwreck (Nuckton et al. 2002).

Apart from body fat, also the high BMI of 35.6 kg/m^2^ of the swimmer might explain that he maintained body core temperature during ice swimming. In 119 competitors (70 male, 39 female) in a 19.2-km open water swimming race in Perth, Western Australia, a higher BMI was associated with a decreased risk of hypothermia (Brannigan et al. 2009).

Specific anthropometric characteristics such as a high body height and a high body mass (due to high body fat) seem to prevent from dying early in cold water. Keatinge et al. (1986) described a 23-year old Icelander who had been fishing off Iceland when his boat capsized. Water temperature was at 5–6°C and air temperature at −2°C. He and two companions climbed on to the keel but after ~45 min they swam towards the shore, ~5 km away. While his two companions disappeared within 10 min, he was able to swim for 5–6 h to the shore while only wearing a shirt, sweater and jeans. The authors argued that his skin-fold thicknesses enabled him to survive (Keatinge et al. 1986).

Apart from anthropometric characteristics, previous experience in ice swimming and open-water swimming might also be an important aspect for a successful open-water swim (Drygas et al. 2014; Knechtle et al. 2009). The present athlete prepared for about 3 years and documented more than 60 trainings prior to tackle an official ‘Ice Mile’. The successful ice swimmer in the study of Knechtle et al. (2009) took ice baths, completed regular swims in water of 18°C for 40 min over 1.6 km, swam 1 km in water of 1°C in Antarctica and watched movies of ice swimming and ice diving. The duration of the swim might also be of importance. Most probably, the duration of less than 1 h for an ‘Ice Mile’ also prevented from hypothermia. People immersed after shipwreck in water colder than 6°C usually die of hypothermia within 75 min (Molnar 1946). The present data support this. Lowest body core temperature was negatively correlated to swim time and decrease/increase in body core temperature was positively correlated to swim times.

### The lowest body core temperature was recorded after the swims

In both the trainings and the two ‘Ice Miles’, the lowest body core temperatures were recorded after getting out of the water. Afterdrop, defined as continued cooling following removal from cold stress, was investigated in eleven subjects following the ‘New Year’s Day Alcatraz Swim’ held in open water of ~11.7°C (Nuckton et al. 2000). The lowest recorded body core temperatures of all eleven subjects ranged from 34.7 to 36.7°C with a mean of 36.0°C. All subjects with recorded pre-event temperatures had post-event lowest body core temperatures that were lower than the pre-event body core temperatures. On average, the mean decrease in body core temperature was 0.99°C. In nine subjects, body core temperature was measured for 45 min post swim and potential predictors were investigated. The surface/volume ratio and BMI were related to the lowest recorded body core temperature and the duration of the afterdrop. Smaller subjects had lower body core temperatures than larger subjects and the duration of the afterdrop was shorter in smaller individuals (Nuckton et al. 2000).

From a practical point of view for future ice swimmers, the rather high value for the lowest body core temperature between 34.6 and 37.7°C after the swims are not life-threatening. Ventricular fibrillation needing resuscitation seems to occur at a body core temperature of ~32.5°C after immersion in cold water (Harries et al. 1981).

### Limitations of the current work and future research directions

While the addition of core temperature during the swim is insightful, the addition of other increasingly practical thermophysiology measures (for example, skin temperature, respiratory rate, heart rate, oxygen saturation, hydration and perceived thermal sensation) would offer great insight into the demands and risks of ice water swimming.

## Conclusion

This case report shows that an experienced ice swimmer with a high BMI and high body fat percentage suffered no hypothermia during ice swimming. However, body core temperature dropped to <36°C after the swim. Water temperature and body core temperature at the finish of a swim were the most important predictors for the lowest body core temperature measured after the finish of a swim. Future athletes intending to swim an ‘Ice Mile’ should be aware that a large body fat percentage prevents from suffering hypothermia during the swim, but not after the swim.
